# Pelvic Floor Disorders Due to Anal Sexual Activity in Men and Women: A Narrative Review

**DOI:** 10.1007/s10508-024-02995-2

**Published:** 2024-09-17

**Authors:** Avital Bar Chen, Leonid Kalichman

**Affiliations:** 1 Department of Physiotherapy, Recanati School for Community Health Professions, Faculty of Health Sciences, Ben Gurion University of the Negev, P.O.B. 653, 84105 Beer Sheva, Israel; 2 Meuhedet Health Services, Central District, Tel Aviv, Israel

**Keywords:** Anal intercourse, Pelvic floor dysfunction, Anodyspareunia, Fecal incontinence, Pelvic floor physical therapy

## Abstract

Recent evidence shows that consensual anal penetrative intercourse has become more prevalent, not only limited to gay, bisexual, and other men who have sex with men but also for women who are in a sexual relationship with men. The aim of this review was to study the influence of consensual anal intercourse on pelvic floor function and the role of pelvic floor physical therapy treatment in preventing or treating consensual anal intercourse-induced anodyspareunia and/or fecal incontinence. We reviewed 68 references that showed that anal penetrative intercourse is a risk factor for anodyspareunia and fecal incontinence in both men and women. This risk of anal intercourse may increase with emotional discomfort, an overactive pelvic floor, lack of lubrication, frequency of anal penetrative intercourse, and hard practice (BDSM: bondage and discipline, dominance and submission, sadism, and masochism). It seems that pelvic floor physical therapists play an essential role in preventing and treating pelvic floor dysfunctions due to anal intercourse, which can lead to anodyspareunia and fecal incontinence; the treatment includes education, pelvic floor training with and without biofeedback, electric stimulation, manual therapy, and dilatators. Further studies are warranted to enhance our understanding of the causes and treatment efficacy of pelvic floor dysfunctions due to anal penetrative intercourse.

## Introduction

The terms anal sex and anal intercourse are commonly used synonymously to refer to a dyadic sex act involving inserting and thrusting one partner’s penis into the anus of the other (McBride & Fortenberry, 2010). However, it does not always mean penetration with a penis; sex toys, fingers, or a tongue can achieve penetration. Although in the past, anal penetrative intercourse was attributed mainly to gay male activity, people of all sexual orientations and gender identities engage in anal sex (Brennan, 2020). Anal sex is an ancient and persistent taboo, a long-stigmatized sexual practice, and occasionally not considered "sex" (Sanders & Reinisch, 1999). Recent evidence shows that anal penetrative intercourse has become more prevalent during the past two decades and is not limited only to gay men.

Recently, research has shown that the practice of anal penetrative intercourse is increasing among men and women in sexual relationships in the USA and the UK (Leichliter, 2008). The prevalence of men and women anal intercourse varies by age and subgroups. Previous studies have estimated that 25–44% of men who are in a sexual relationship with women and 16–36% of women who are in a sexual relationship with men have engaged in anal sex (Habel et al., 2018). The percentage of anal intercourse between gay men and men who have sex with other men ranges from 42.5 to 82% (Cheng, 2022). For example, a study of Polish men who engage in sexual intercourse with men revealed that receptive anal intercourse was practiced by 82% (Grabski & Kasparek, 2020).

Most studies reporting on anal intercourse or anal sex between gay men, men who have sex with other men or men and women focused on the infectious risks associated with this phenomenon, and only a few have considered the risk of anorectal dysfunction. Anal penetrative sex may cause several anorectal dysfunctions, including fecal incontinence and anodyspareunia (pain occurring due to receptive anal penetrative intercourse (Cheng, 2022)). In this narrative review, we will review the anatomy of the anal canal, the influence of consensual anal penetrative intercourse on the dysfunctions of the pelvic floor, and the role of physical therapy and the means by which pelvic floor physical therapy can help prevent or treat these problems. Although other treatment options exist, we will not discuss those in this review.

## Method

PubMed and Google Scholar were searched from May 2022 to November 2022, with no date restrictions, and only articles written in English were included. Systematic reviews and clinical trials were prioritized. The keywords used were: "anal intercourse" or "anal sex" or "Heterosexual anal intercourse" and "pelvic floor dysfunction, overactive pelvic floor," "anal pain," "anodyspareunia," "anal incontinence," "fecal incontinence," and "physical therapy" or "physiotherapy." We excluded from this review all papers discussing non-consensual anal intercourse and all papers discussing disease-related anal intercourse dysfunction (such as infections, surgeries, irritable bowel syndrome, and cancer). We included 68 references in the final review.

## Results

### Anatomy and Primary Function of the Anal Canal

The pelvic floor, comprising ligaments, fascial tissues, and two main muscle layers, primarily supports the pelvic and abdominal organs (Gentilcore-Saulnier et al., 2010). Pelvic floor function synergy acts with the trunk, providing stability and pain-free movement, supporting viscera, and preventing fecal or urine incontinence (Sapsford et al., 2001). The function and stability of the viscera also depend on the pelvic floor's bones and fascia, as well as muscles and coordination with the diaphragm, the transverse abdominis, and the multifidus muscles. Dysfunction of the joints and muscles surrounding the pelvic floor, i.e., hips, lumbar spine, sacroiliac joints, shortening of the iliopsoas, hamstrings, and hip adductors, can contribute to pelvic pain and may affect the viscera. On the other hand, good breathing quality and the ability to relax the levator ani allow painless sexual penetration (Rosenbaum, 2005).

The anal canal is 2.5–5 cm in length, originating at the levator ani muscle and opening into the anal verge (Barleben & Mills, 2010). The internal and external anal sphincter muscles surround the anal canal. The internal anal sphincter muscle is composed of smooth muscle in continuation with the inner circular power of the rectum and superiorly wrapped by the levator ani muscle. The external anal sphincter muscle radially wraps around the internal anal sphincter and is composed of skeletal muscle in continuation superiorly with the puborectalis and levator ani muscles (Solan & Davis, 2013).

The main pelvic floor dysfunctions described in the literature are Anodyspareunia; defined as pain occurring during and after receptive anal penetrative intercourse (Cheng, 2022), and fecal incontinence; defined as at least once involuntary loss of solid stool, mucus, or liquid during the last 30 days but does not include gas (Whitehead et al., 2009).

### Anodyspareunia: Definition, Prevalence, and Risk Factors

Anal intercourse has been reported as painful by some women, especially the first time, although others have described more pleasurable experiences (McBride, 2019). The physiological factors that make receptive anal penetrative intercourse painful include the type of epithelium found in the anus, anal sphincter tension, the lack of natural lubrication, and the anorectal angle (Grabski & Kasparek, 2020). When women were asked about probable causes of the pain during anal intercourse, most stated that they could not sufficiently relax (Stulhofer & Ajduković, 2013). A study of Polish gay, bisexual, and straight men found that 77.7% of the men who practiced anal penetrative intercourse experienced some degree of pain, 44.3% felt slight pain, 57% moderate to very strong, and 10% experienced strong and very strong pain (Grabski & Kasparek, 2020).

In Herbenick et al. (2015) study, the authors reported that approximatly 15% of men who are in a sexual relationship with women and 72% of women who are in a sexual relationship with men engaging in anal intercourse reported some degree of pain during their most recent sexual experience that involved penile-anal intercourse. For men, anal intercourse with a female partner generally involves anal penetration by fingers, sex toys, or other objects. Men who reported pain described it as "slightly painful." Among the women, 25.6% said that their anal intercourse experience was "slightly painful," 38.5% as "moderately painful," 2.6% as "quite a bit painful," and 5.1% as "extremely painful."

In three different age groups of women, 18–24, 30–39, and 70 + , 100% of the women reported some pain during anal intercourse. There was no association between pain during anal intercourse and the ages of the men or women; 53% of men and 63% of women reported pain during anal intercourse stated that the pain lasted less than 5 min. However, 25.5% of the men claimed that the pain they experienced lasted more than an hour, and 14.5% stated that it lasted a day or more. The two most common pain sites in men during receiving anal intercourse are on their penis and inside their urethra. Some felt it at the anal opening, scrotum, pelvic, and abdominal area, or inside the rectum. Most women described their pain at the anal opening (85.53%), and some reported pain inside their rectum (Herbenick et al., 2015).

In Grabski and Kasparek’s (2020) study on sexual anal pain in men, the authors found a statistically significant relationship between anodyspareunia and internalized homophobia. Inner discomfort causes emotional discomfort, thus preventing a sexual response and relaxation. Furthermore, anodyspareunia was found to correlate with performance anxiety, low sex drive, and anorgasmia. Yet, age, steady relationships, sexual experiences, and physical activity were all found to be negatively associated with anodyspareunia. Men (25%) suffering from anodyspareunia reported that their pain was associated with psychological factors, penis size, and lack of finger-anus foreplay. However, less than 25% said that the lack of lubrication and sexual arousal caused the pain (Damon & Rosser, 2005). Despite the rising prevalence of anal sex and therefore rising prevalence of anodyspareunia, there is no direct distinction of anodyspareunia even in the latest ICD-11 (World Health Organization, 2019), although it may be classified under HA20 as Sexual Pain-Penetration Disorder. Additionally, it may be classified under the HA2Y unit as other specified sexual pain disorders, which can potentially be used to classify people experiencing anal dyspareunia, according to Plewka et al. (2023).

### Fecal Incontinence

Normal defecation relies upon the ability of the anorectum to discriminate between the states of fecal matter: solid, liquid, or gas. Continence also depends on voluntary and involuntary control (Barleben & Mills, 2010) and is determined by the number of reflexes, the function of the anal sphincters at rest, and the closing pressure at all times unless the person needs to defecate. Subsequently, the anal sphincter relaxes (Palit et al., 2012). Atrophy or weakening of the external anal sphincter and internal anal sphincter are common causes of fecal incontinence, in addition to the ability of the external anal sphincter to contract reflexively during the increase of intraabdominal pressure, i.e., sneezing (Remes‑Troche & Rao, 2008).

Fecal incontinence is a condition that can affect the quality of life, i.e., social embarrassment, isolation, and loss of employment (Bharucha et al., 2006; Norton, 2004). Risk factors associated with fecal incontinence are age, comorbid diseases, depression, and stool consistency (Markland et al., 2016). The prevalence of fecal incontinence in the population is poorly documented and reported to be more common in older adults (Markland et al., 2016). A study by Whitehead et al. (2009) reporting on the prevalence and risk factors of fecal incontinence in adults residing in the USA found a prevalence of 8.3% in non-institutionalized adults who reported fecal incontinence at least once during the last 30 days. Fecal incontinence was found to be equally common in women and men. For both sexes, prevalence increased from 2.6% at 20–29 years of age up to 15.3% at > 70. Vaginal deliveries increase the risk of fecal incontinence from 5.9% in women who have not delivered vaginally to 15.1% in women who have undergone four or more vaginal deliveries. Furthermore, chronic illnesses have been found to be a risk factor for women but not for men.

Ilnyckyj’s (2010) Canadian study reporting on fecal incontinence sampled 361 men and 366 women (mean age, 47). The prevalence of fecal incontinence was 2% in the entire population and 3.7% if a physician-diagnosed gastrointestinal illness was added. In a study by Lim et al. (2014), the prevalence of fecal incontinence in Singapore was 4.7%. The authors found a statistically significant association between fecal incontinence and females, with women being three times more likely to suffer from fecal incontinence than men. In addition, fecal incontinence significantly increased with age. Participants aged ≥ 53 years were more likely to suffer from fecal incontinence compared with their younger counterparts.

Garros et al. (2021) studied the prevalence of fecal incontinence in 21,762 men who had performed anal intercourse with men. The participants answered an online survey relating to stool leakage: "During the last month, have you experienced any involuntary leakage of stools?"; 8% answered that they suffer from fecal incontinence. The mean age of the population was 35.3 years (95% confidence interval (CI) [34.8–35.2]). Those declaring fecal incontinence were older (median age: 38.5 years [26–50] vs. 32.0 [24–45); *p* < .001). The researchers found that the prevalence of fecal incontinence within the month prior to the survey increased significantly with the frequency of anal intercourse (12.7% for subjects practicing anal intercourse more than once a week compared to 5.7% for subjects who did not, *p* < .001). The prevalence of fecal incontinence was also significantly higher among those practicing chemsex (use of psychoactive drugs during sex) (21.4% vs. 7.2%) and hard practices (BDSM: bondage and discipline, dominance and submission, sadism, and masochism) during anal sex (18.1% vs. 7.2%).

Markland et al. (2016) surveyed 2100 men and 2070 women aged 20–69 years as to their sexual behavior and fecal incontinence; 37.3% of the women reported engaging in anal intercourse. A higher number of the women reported engaging in anal intercourse was found among younger women aged 20–49; 4.5% of the men reported engaging in receiving anal intercourse. Fecal incontinence rates were higher among the women (8.3% (CI 6.9–10.0%)) versus the men (5.6% (CI 4.3–7.2%)) who reported performing anal intercourse compared with those who did not. Anal intercourse was significantly associated with prevalent fecal incontinence among men and women. Additionally, men who engaged in anal intercourse at least once in their lifetime reported a higher prevalence of fecal incontinence than men who did not engage in lifetime anal intercourse.

An e-mail survey of 1003 women regarding their sexual practices and the effects on their bowel and bladder symptoms found that 12% performed anal intercourse as part of their sexual life, and 32% had engaged at least once. The authors also reported that 17% of the women who performed anal intercourse reported suffering from vaginal dyspareunia; 28.3% of women who performed anal intercourse during the previous month reported fecal incontinence, there is also association between fecal incontinence and a history of anal sphincter tears and chronic bowel infection (Geynisman‑Tan et al., 2018).

Anal intercourse between gay men at a frequency of only once a week was not found to be associated with an excess risk of fecal incontinence. On the other hand, a higher frequency of anal intercourse (at least several times per week) was significantly associated with fecal incontinence (Garros et al., 2021). Manual anal sexual practices, i.e., fisting (manual–anal intercourse), were found to be considerably associated with fecal incontinence (Garros et al., 2021). One of the explanations for the association between anal intercourse and fecal incontinence can be found in a study involving 40 gay men. The researchers found a significant reduction in both maximum anal resting pressure and anal mucosal electrosensitivity in subjects who performed anal intercourse compared with subjects who did not (Miles et al., 1993). It was found that anal dilatation can cause a reduction in resting anal pressures and disrupt the internal and external anal sphincter (Speakman et al., 1991).

### *Colon* and Rectal Perforation

Manual–anal intercourse ("fisting") is a form of sexual practice, an act of inserting a closed hand, part, or all of the forearm, or both fists into the rectum, which can cause anodyspareunia. There is also evidence of colon perforation secondary to this sexual practice. Spears et al. (1995) reported a case of a 42-year-old man who, after manual anal intercourse, was admitted to the emergency unit complaining of abdominal pain and anal bleeding, requiring immediate medical treatment. Another case report described a 20-year-old woman who had anal intercourse with penile penetration and suddenly experienced an acute onset of severe pelvic pain (Symeonidis et al., 2015). She suffered from a 4-cm tear on the posterior vaginal fornix, which had penetrated the rectal lumen. The injury did not involve anal sphincters or the perineum.

Another case study described a 38-year-old woman who, after penile-anal intercourse, complained of sudden bleeding and anal pain (Albertsen & Christensen, 2017). She was diagnosed with an anovaginal fistula without anal sphincter injury. It is important to note that a rectovaginal fistula without injury to the anal sphincter due to consensual penile–anal intercourse is very rare. This phenomenon has been described only twice in the literature.

### Treatment for Anal Sex-Related Pelvic Floor Dysfunction, Particularly the Role of Physical Therapy

Sexual dysfunction may derive from several factors, including physical, psychological, sociocultural, and interpersonal factors. Therefore, an approach involving a multidisciplinary team (including medical doctors, psychologists, sex therapists, and physical therapists) is important for optimal treatment (Assmann et al., 2022). Here, we reviewed only the role of pelvic floor physical therapy in treating anodyspareunia and fecal incontinence.

Research reporting on an overactive pelvic floor in women and men practicing anal intercourse is scarce. The studies mainly describe dyspareunia and the pain felt with vaginal penetration. The treatment goals of dyspareunia induced by an overactive pelvic floor are: to increase the awareness and proprioception of the pelvic floor muscle, improve the relaxation ability of the pelvic floor muscle, decrease the tone of the pelvic floor muscle, relieve potential trigger points, increase blood flow to the pelvic floor muscle, reduce fear and anxiety toward penetration, and encourage relaxed penetration (Bergeron & Lord, 2010).

The treatment includes several therapy options: manual (digital intravaginal techniques, soft tissue mobilization, stretching, and desensitization), insertion techniques using dilatators, pelvic floor electromyographic (EMG) biofeedback, electrical muscle stimulation, education, and home exercise (Gentilcore‑Saulnier et al., 2010). It is possible that the same goals and treatment could be applied to treat anodyspareunia with a manual approach through the anus instead of the vagina. Typical physical therapy treatments for fecal incontinence comprise patient education, pelvic floor, and muscle training with or without biofeedback, electrostimulation, and manual therapy techniques (Scott, 2014). In combination with conservative treatment, pelvic floor muscle training is recommended as a first-line treatment for patients with fecal incontinence (Ussing et al., 2019).

A physical therapy session for anal sex-related pelvic floor dysfunction includes paramedical anamneses, which include gathering data about the patient’s daily activities, family and personal history, and history of abdominopelvic symptoms (González‑Castro et al., 2023). In addition, an inspection of position, locomotion, mobility of the trunk, and breathing techniques. Patients are familiarized with the anatomy and physiology of the pelvic organs and muscles using photographs and anatomical models. During the treatment session, exercise instructions are given, and pelvic floor muscles are palpated (Cornel et al., 2005).

### Prevention and Education

Education is an essential tool for the prevention of anal intercourse-induced pelvic floor dysfunctions. An explanation of the anatomy and physiology of the pelvic organs and muscles using appropriate photographs and anatomical models is essential to decrease anxiety (Cornel et al., 2005). Anal intercourse can be painful; therefore, explaining lubrication and relaxation of the pelvic floor muscles is also essential (Grabski & Kasparek, 2020). Lifestyle and behavioral modifications and education regarding healthy bowel and bladder habits often help patients minimize the factors that aggravate their symptoms (Bradley et al., 2017). Furthermore, it is essential to discuss possible dysfunctions such as pain and incontinence as potential risks of receptive anal intercourse and the need for professional medical evaluation and treatment (Cleveland Clinic, 2022).

### Pelvic Floor Muscle Training

Therapeutic exercises help normalize muscle length, strength, and function. Exercises for postural correction and muscle flexibility can reduce strain across the lower trunk, pelvic girdle, and intrinsic hip muscles. Although widespread, pelvic floor muscle contractile exercises (known as Kegel exercises that are used for muscle strength, power, and relaxation) are not always warranted, especially in cases of an overactive pelvic floor. Core training may cause the shortening of pelvic floor muscles and, therefore, generate pain. Hence, it may need to be suspended until muscle flexibility and length have improved (Bradley et al., 2017). Pelvic floor muscle training improves the muscles' function by enhancing contraction, endurance, and coordination. Nevertheless, muscles must be trained to be flexible and mobile (Rosenbaum & Owens, 2008). Specific breathing techniques can also be used first to release anxiety, which reduces pain (Busch et al., 2012), and second, during aspiration, pelvic floor muscles relax (Park & Han, 2015).

In cases of fecal incontinence, pelvic floor muscle training can improve endurance, tension, power, and coordination of the anal sphincter and the pelvic floor muscles (Scott, 2014). Repetitive voluntary contractions of the external anal sphincter have been used to improve fecal incontinence (Mei et al., 2021). Repetitive anal squeeze exercises against a resistive load effectively improve the strength of the external anal sphincter (Patel et al., 2021).

### Biofeedback

EMG biofeedback training is a physiologic process. Contraction and relaxation of a muscle are converted to a visual or auditory signal that is then fed back to the patient; the goal is to learn from the feedback how to control dyssynergia of the muscle (dyssynergia is a disruption of normal coordination of muscle movement), thus improving coordination (Hite & Curran, 2021). Biofeedback has been used to improve the overactive pelvic floor in many conditions, i.e., using pressure measurements (manometry) or EMG biofeedback activity within the anal canal to teach patients with dyssynergia pelvic floor to relax the pelvic floor muscles when straining to defecate (Simón & Bueno, 2009). Biofeedback therapy significantly decreases the anismus index (anismus is a dyssynergy of defecation), pelvic floor muscles resting amplitude, and difficult and painful defecation (Ahadi et al., 2014).

Men with chronic pelvic pain experience a pathological tenderness of the striated pelvic floor muscles and pelvic floor function (Zermann et al., 1999). Cornel et al. (2005) studied 31 men suffering from chronic pelvic pain who were trained in a pelvic floor biofeedback re-education program and found that the mean value of the pelvic muscle tonus significantly decreased after training. It was found that women with vulvar pain trained with EMG biofeedback reported reduced pain sensation or were pain-free by the end of the treatment protocol (Morin et al., 2017); 93.3% of the entire sample were pain-free after sexual intercourse after 6 months of training (McKay et al., 2001). Biofeedback is also one of the techniques for learning and facilitating pelvic floor muscle exercises (Duelund‑Jakobsen et al., 2016), helping to isolate appropriate pelvic floor muscles, anal sphincter contractions and improving fecal incontinence (Sigurdardottir et al., 2020). Research has shown that biofeedback improves fecal incontinence symptoms in 75% of cases (Lacima et al., 2016) and can help when there is damage to the sphincters (Sigurdardottir et al., 2020).

### Electrical Stimulation

Electrical stimulation is another modality available to the physical therapist in improving the posterior pelvic floor muscles, thereby enhancing the strength, speed, and endurance of the voluntary anal sphincter contraction and heightening the sensation of the anal sphincter. The use of electrical stimulation can assist in cases of fecal incontinence by straightening the anal muscle contraction (Hosker et al., 2007). In a study of women with dyspareunia, it was found that 95% of women with chronic tension and hypertonicity of the pelvic floor muscles had less pain, achieved a complete resolution of pelvic floor hypertonicity, and a reduction of symptoms after 10 sessions of transcutaneous electrical nerve stimulation (TENS) (Dionisi & Senatori, 2011).

### Manual Therapy

Many studies have reported on the efficacy of therapeutic massage using techniques such as deep tissue massage, myofascial mobilization, and trigger point release. Massage therapy acts as a pain reliever (Koren & Kalichman, 2018). Shortening and increasing the tone of the pelvic floor muscles is linked to myofascial pain syndrome (Itza et al., 2013). Most research studies have reported on manual therapy of the pelvic floor and dyspareunia. In Silva et al.’s (2017) randomized control trial, women underwent a transvaginal massage using the Thiele technique. The Thiele technique is a massage of the pelvic floor muscles that begins from the point of origin to insertion, with a bearable pressure over a period of 5 min. The results showed a significant improvement in dyspareunia after four treatments for 4 weeks. The improvement of the dyspareunia lasted at least 24 months. In another study regarding transvaginal massage, given twice a week for 5 weeks, the researchers found improvement in irritable bladder symptoms in interstitial cystitis cases and decreased pelvic floor muscle tone. The improvement was preserved for 4.5 months (Oyama et al., 2004). In a randomized control trial, symptom severity and frequency of urological chronic pelvic pain syndrome decreased after 10 weekly treatments using a transvaginal or transrectal treatment that included soft tissue manipulation of the pelvic floor, periurethral connective tissues, arcus tendinous, and fascia pelvis. No follow-up was conducted to evaluate the long-term effects (Fitzgerald et al., 2013). Ghaderi et al. (2019) described a 3-month, once-a-week treatment for dyspareunia. Manual techniques were used to release trigger points in the pelvic floor by using myofascial soft tissue release and deep intravaginal massage. There is evidence that massaging the anal sphincter (in addition to dilatation) with a finger helps to achieve relaxation of the hypertonic anal sphincter in cases of an acute anal fissure, this improvement lasted for 6 months (Gaj et al., 2017). Self-anal massage also decreases anxiety and muscle tension if preformed before anal intercourse (Vansintejan et al., 2013). Anderson et al. (2006) reported a significant improvement in chronic pelvic pain syndrome in men treated by releasing the trigger points in the pelvic floor muscles with applied pressure and relaxation exercises. The men were treated weekly for 4 weeks, followed by 8 weeks of biweekly treatments. The improvement was preserved in the 1-month and 5-month follow-ups.

### Dilatators

Anal dilatators induce relaxation of the hypertrophic, hypercontracted anal sphincter, and are a recommended treatment method for anal fissures (Gaj et al., 2017). Commercially available anal stretching devices of different diameters and lengths were found effective in improving chronic prostatitis and chronic pelvic pain syndrome (Itza et al., 2013). For dyspareunia, it is recommended to use vaginal dilators to help relax the pelvic floor, reduce pain, and overcome fear (Morin et al., 2017).

## Discussion

The objectives of this study were to review the literature relating to the effects of consensual anal penetrative intercourse on women's and men's pelvic floor function and how pelvic floor physical therapists may prevent and treat these problems. Anal penetrative sex is common among gay men, bisexuals, other men who have sex with men, and men and women in sexual relationships; 25–44% of men who are in sexual relationships with women and 16–36% of women who are in sexual relationships with men engage in anal intercourse (Habel et al., 2018). Anal intercourse is more common in younger women (Markland et al., 2016). Within the gay community, approximately 82% engage in anal intercourse (Grabski & Kasparek, 2020). Unfortunately, there is no data related to anal intercourse among women who are in sexual relations with women and with other genders or sexual orientations.

Anal penetrative intercourse, in most cases, is safe and pleasurable and, over the years, has become more prevalent. Nevertheless, in some cases, anal penetrative intercourse may cause two main pelvic floor dysfunctions: anodyspareunia and fecal incontinence. Anal penetrative intercourse may sometimes cause anodyspareunia, mainly due to an overactive pelvic floor and emotional discomfort (Grabski & Kasparek, 2020). These dysfunctions may also be associated with a lack of lubrication and penis size (Damon & Rosser, 2005); 15% of men who are in sexual relationships with women and 72% of women engaging in anal intercourse have experienced some degree of pain (Herbenick et al., 2015). Some women stated that they could not sufficiently relax during anal intercourse, which may contribute to anodyspareunia (Stulhofer & Ajduković, 2013). It has been widely reported that women who engage in vaginal intercourse, not for their self-pleasure but for other reasons, report more vulvar pain during intercourse (Corsini-Munt et al., 2020; Dubé et al., 2017; Rosen et al., 2015). It has also been reported that only one-third of women who engage in anal intercourse do so for their pleasure (Geynisman‑Tan et al., 2018); therefore, engaging in anal intercourse, not for pleasure but for other reasons, might increase the inability to relax the muscles, which contributes to anodyspareunia.

Pelvic floor treatment includes education on pelvic floor anatomy, function, use of lubrication (Grabski & Kasparek, 2020), and healthy bowel and bladder habits (Bradley et al., 2017). Education reduces anxiety and helps relax the pelvic floor (Grabski & Kasparek, 2020). Pelvic floor muscle training improves coordination, flexibility, and mobility (Rosenbaum & Owens, 2008). Pelvic floor treatment can be conducted with or without biofeedback. Biofeedback treatment improves muscle coordination and control (Hite & Curran, 2021), teaching patients how to relax their pelvic floor muscles. After training, the pelvic muscle tone significantly decreases (Cornel et al., 2005).

Electrical stimulation is another modality available to the pelvic floor physical therapist for anodyspareunia treatment. A session of TENS reduces the hypertonicity of the pelvic floor muscles, thus reducing pelvic floor pain (Dionisi & Senatori, 2011). Manual pelvic floor therapy includes massaging the muscles, trigger points, and myofascial soft tissue release, decreasing pelvic floor muscle tone, reducing pain, and decreasing anxiety (Oyama et al., 2004). Manual release may help achieve relaxation of the hypertonic anal sphincter (Gaj et al., 2017). An additional modality to reduce pain during anal intercourse is the use of dilatators. Dilatators induce relaxation of the hypertrophic and hypercontracted anal sphincter (Gaj et al., 2017), thus reducing anodyspareunia.

Fecal incontinence is another risk factor of penetrative anal sex induced by anal dilatation, disruption of the external and internal anal sphincter, and a significant reduction in anal resting pressure (Miles et al., 1993). Researchers found that the prevalence of fecal incontinence increases with age, frequency of engaging in anal sex (once a week or less was not found to increase prevalence), and hard practice (Garros et al., 2021). In women, fecal incontinence is significantly associated with anal intercourse. Almost 30% of women who engaged in anal intercourse within the previous month reported fecal incontinence (Geynisman‑Tan et al., 2018); 8% of the men reported fecal incontinence (Garros et al., 2021). Men who engaged in anal intercourse at least once in their lifetime reported a higher prevalence of fecal incontinence than men who had not (Markland et al., 2016); 12.7% of men who engaged in anal intercourse more than once a week suffered from fecal incontinence (Garros et al., 2021).

Pelvic floor treatments for fecal incontinence include an explanation of the anatomy and physiology of the pelvic organs and muscles (Cornel et al., 2005), education as to healthy bowel and bladder habits (Bradley et al., 2017), recommendations as to reducing the frequency of engaging in anal intercourse, and pelvic floor muscle training. Pelvic floor muscle training, with or without biofeedback, is used to improve endurance, tension, and power of the anal sphincter and the pelvic floor muscles (Scott, 2014). Repetitive voluntary contractions of the external anal sphincter, mainly against a resistive load, have been used to strengthen the external anal sphincter and improve fecal incontinence (Mei et al., 2021; Patel et al., 2021). Electrical stimulation improves the strength, speed, and endurance of the anal sphincter contraction and sensation and, therefore, may help in cases of fecal incontinence (Hosker et al., 2007).

### Limitations

We are aware that narrative reviews have more potential for bias than systematic reviews. Our search strategy was not systematic; however, we endeavored to conduct a comprehensive search. The studies' quality was assessed and considered using the PEDro score (De Morton, 2009), but no studies were excluded based on quality. Our review was limited to English-language articles, which could constitute some bias.

### Conclusion

A link exists between emotional discomfort, an overactive pelvic floor, the number of times anal intercourse was performed, and pelvic floor dysfunction. The pelvic floor physical therapist endeavors to prevent pelvic floor dysfunction due to anal intercourse, which can lead to anodyspareunia and fecal incontinence. They also provide a safe and open environment for asking intimate questions and receiving counsel. Behavioral treatment and education are common tools in treating the majority of anodyspareunia cases and fecal incontinence induced by anal penetrative intercourse. If complaints of anodyspareunia or fecal incontinence persist, the pelvic floor physical therapist utilizes additional modalities of treatment: manual therapy, pelvic floor muscle training with or without biofeedback, electrical stimulation, and dilatators.

It is essential to continue this type of research, as many questions remain unanswered. For example, why are women more prone to fecal incontinence, and for how long does fecal incontinence continue? Randomized controlled trials evaluating the effect of pelvic floor physical therapy on anal penetrative intercourse-induced anodyspareunia and fecal incontinence in both men and women were not found during our literature review.
